# Supplementary material for: Application of a Two-Dimensional Mapping-Based Visualization Technique: Nutrient-Value-Based Food Grouping
Source: Nutrients. 2023 Dec 4;15(23):5006. doi: 10.3390/nu15235006 (PMC10707954; doi:10.3390/nu15235006)
Supplement: Supplementary file 1 [file nutrients-15-05006-s001.zip › nutrients-2704747-supplementary.pdf]

Supplementary Materials

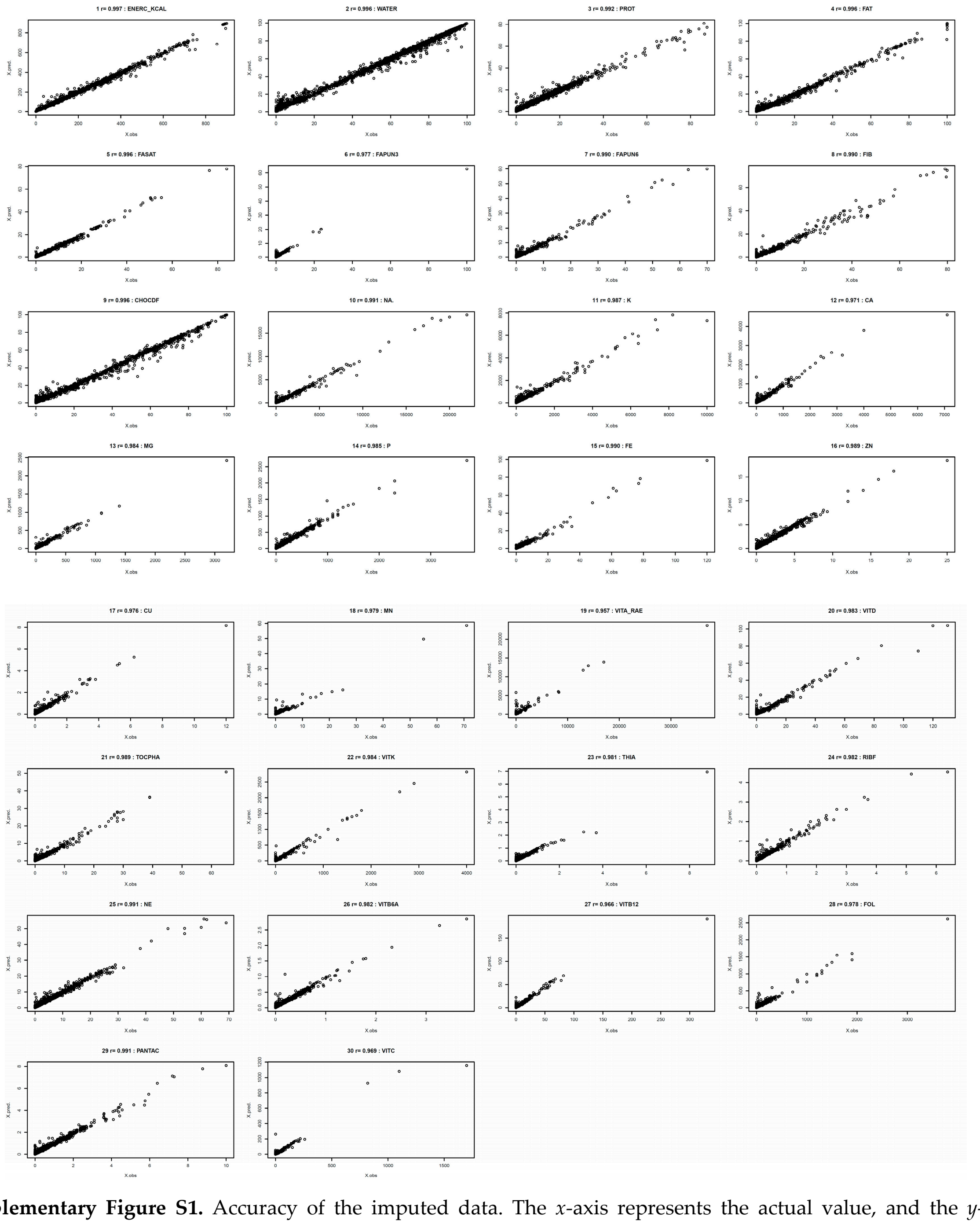

Supplementary Figure S1. Accuracy of the imputed data. The *x*-axis represents the actual value, and the *y*-axis represents the predicted value. For all nutrients, the correlation coefficients between the actual and predicted values were greater than 0.9. The abbreviations are following; ENERC_KCAL: energy, WATER: water, PROT: protein, FAT: fat, FASAT: saturated fatty acid, FAPUN3: n-3 polyunsaturated fatty acid, FAPUN6: n-6 polyunsaturated fatty acid, FIB: dietary fiber, CHOCDF: carbohydrate, NA: sodium, K: potassium, CA: calcium, MG: magnesium, P: phosphorus, FE: iron, ZN: zinc, CU: copper, MN: manganese, VITA_RAE: vitamin A (retinol activity equivalents), VITD: vitamin D, TOCPHA: vitamin E (alpha-tocopherol), VITK: vitamin K, THIA: vitamin B_1, RIBF: vitamin B_2, NE: niacin equivalent, VITB6A: vitamin B_6, VITB12: vitamin B_{12}, FOL: folic acid, PANTAC: pantothenic acid, VITC: vitamin C.
